# Composite Zn(II) Ferrocyanide/Polyethylenimine Cryogels for Point-of-Use Selective Removal of Cs-137 Radionuclides

**DOI:** 10.3390/molecules26154604

**Published:** 2021-07-29

**Authors:** Irina Malakhova, Yuliya Parotkina, Marina Palamarchuk, Marina Eliseikina, Aleksandr Mironenko, Alexey Golikov, Svetlana Bratskaya

**Affiliations:** 1 Institute of Chemistry, Far Eastern Branch of Russian Academy of Sciences, 159 Prosp. 100-Letiya Vladivostoka, 690022 Vladivostok, Russia; newira94@gmail.com (I.M.); azarova.87@mail.ru (Y.P.); marina_p@ich.dvo.ru (M.P.); almironenko@gmail.com (A.M.); glk@ich.dvo.ru (A.G.); 2 A.V. Zhirmunsky National Scientific Center of Marine Biology, Far Eastern Branch of Russian Academy of Sciences, 17 Palchevskogo Street, 690041 Vladivostok, Russia; meliseikina@yandex.ru

**Keywords:** polyethyleneimine, cryogel, point-of-use water treatment, sorption dynamics, cesium, ferrocyanides, RCD model

## Abstract

The feasibility of several approaches to the fabrication of monolith composite cryogels containing transition-metal ferrocyanides for Cs^+^ ion uptake has been evaluated. Although in the series of investigated metal ion precursors (Cu(II), Zn(II), Ni(II), and Co(II)), in situ formation of the sorption active phase in polyethyleneimine (PEI) cryogel was feasible only in the case of Zn(II) ferrocyanide, this approach has shown significant advantages over the immobilization of ex situ synthesized ferrocyanide nanoparticles. Nanoparticles of the mixed ferrocyanide Zn_1.85_K_0.33_[Fe(CN)_6_] formed in situ had an average size of 516 ± 146 nm and were homogeneously distributed in the monolith located at the polymer surface rather than embedded in the matrix. The Young modulus of the PEI cryogel increased after modification from 25 to 57 kPa, but composites maintained high permeability to the flow. Sorption of Cs^+^ ions has been investigated at superficial velocity up to 8 m/h. Steep breakthrough profiles and uptake efficiency of >99.5% until breakthrough point confirmed that a supermacroporous structure of the monolith composite assured good mass transfer, so that intraparticle diffusion was not the limiting stage of sorption kinetics. Application of the rate-constant distribution model (RCD model) to analyze the breakthrough curves of Cs^+^ sorption allowed the identification of two types of sorption sites with a difference in sorption rate constants of ~1 log unit. Most likely, sorption on “fast” sorption sites was governed by ion exchange between Cs^+^ ions in solution and K^+^ ions in the ferrocyanide lattice. Cs-137 radionuclide removal was investigated using the monolith composite columns of various geometries at superficial velocity up to the 6.6 m/h; specific gamma activity was reduced from 265 kBq/L to the background level, showing high potential of these materials for POU application.

## 1. Introduction

The aging of water distribution networks, increasing costs for water transportation and the need for alternative water sources and wastewater reuse, has significantly promoted the development of high-performance small-scale point-of-use (POU) systems [1] with porous ceramic [2] or polymeric [3] filters. Although many materials for POU technologies have been targeted at water disinfection, composites containing highly selective inorganic sorbents in the polymer matrices are of great interest for specific POU applications, e.g., the removal of highly toxic pollutants, such as Hg(II) [4,5] and As(III)/As(V) [6] in gold-mining areas or Cs^+^ radionuclides in areas exposed to nuclear accidents [7,8].

Different types of polymer–inorganic sorbents for cesium uptake, including porous sponges for collecting radioactive spills [7], have been reported recently [7,8,9,10,11]. In most cases, porosity, which is crucial for good sorption kinetics, is achieved via freeze-drying of the polymer solutions containing dispersed inorganic nanoparticles, e.g., transition-metal ferrocyanides, which are well known as highly selective sorbents for cesium ions [12]. Despite good performance under static conditions, freeze-dried composite materials containing mixed nickel–potassium ferrocyanide were not very efficient in the form of porous discs packed as a stack in the sorption columns, due to mass transfer limitations, meaning that recirculation of the solution at high flow rate was necessary to reach the target decontamination factor [11].

As shown earlier [13], due to the different flow conditions and the entrapment of the solution inside the highly swellable supermacroporous matrix, the sorption kinetics on porous beads in fixed bed is notably worse than the kinetics on monoliths of the same materials [13,14]. This explains why sorption on freeze-dried discs was efficient only under high flow rates, forcing the solution to go through the porous structure [11]. This problem can be eliminated by the fabrication of macroporous composite monoliths directly in the sorption column. In this case at all flow rates, all sorption sites will be equally accessible, and mass transfer will be limited only by diffusion into the polymer walls, which usually have a thickness of up to 10 µm. A large size of pores (dozens or hundreds µm) is also crucial for composite monoliths to avoid flow blockage due to the presence of inorganic particles and their aggregates. Cryogels, which can be obtained via the polymerization of monomers or cross-linking of water-soluble polymers under subzero temperature [15], look to be very attractive candidates to meet these requirements for POU application.

In most cases, beads or monoliths of composite sorbents are obtained by dispersing ex situ synthesized nanoparticles in a monomer or polymer solution followed by polymerization [6,16], cross-linking, or freeze-drying [7,17,18]. However, when a polymer matrix can efficiently bind metal ions—precursors of the selective sorbents—the inorganic phase can be formed in situ in a pre-shaped monolith [19]. Regardless of the method of composite fabrication, the presence of the inorganic phase affects the swelling, stiffness [17], and, thus, hydrodynamic properties [16] of the monoliths, meaning that fine-tuning of the composition, i.e., selection of an appropriate polymeric matrix [20] and optimization of cross-linking density and loading degree [16,17], must be important for the overall performance of the cryogel sorbents. Unfortunately, there are only a few examples of composite cryogel application under dynamic conditions [6,11,17], which however illustrate that maximal decontamination factors reached in batch at equilibrium are often higher than in a fixed bed at reasonable flow rate. Thus, the optimization of the composite monolith fabrication method requires great effort and is not straightforward.

Recently, we reported on the fabrication of the composite cryogel N-(2-carboxyethyl)chitosan/Co(II) ferrocyanide for Cs-137 uptake [19], which showed high efficiency in a fixed bed up to a flow rate of ~40 bed volumes (BV)/h; however, application at higher flow rates was limited due to low Young modulus (6 ± 2 kPa) and compression of the monolith. Using polyethyleneimine (PEI) cryogel as a matrix for in situ formation of Cu(II) ferrocyanide, we failed to obtain an efficient sorbent for cesium ions, despite good mechanical properties of the composite [20]. We suggested that the high stability of the Cu(II) complex with PEI(II) prevents the formation of Cu(II) ferrocyanide nanocrystals, which are responsible for the selective sorption of cesium ions.

Here, we evaluate the feasibility of different approaches to fabricate composite PEI cryogels containing transition-metal ferrocyanides and report on optimized composition and fabrication method to obtain monoliths applicable in POU technology for the removal of cesium radionuclides.

## 2. Results and Discussion

### 2.1. Fabrication of the Composite Cryogels Containing Transition-Metal Ferrocyanides

**Figure 1:**
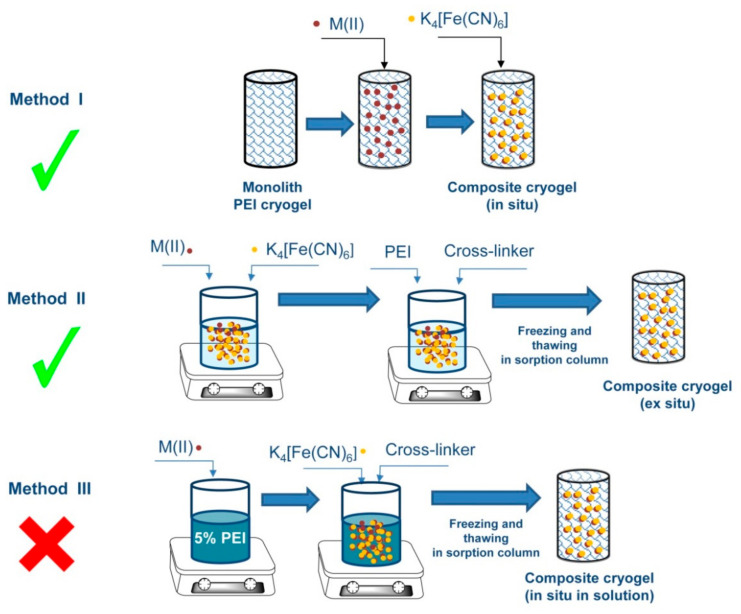
Schemes of the composite cryogel fabrication.

Taking into account our earlier negative experience with the fabrication of composite PEI cryogel for cesium ion sorption via the sequential loading of PEI monolith with Cu(II) and [Fe(CN)_6_]^4−^ ions [20], and the assumption that weaker binding of precursor metal ions to PEI will be beneficial for the fabrication of the composite cryogel with good selectivity to cesium ions, we have tested the same approach (Method I, Figure 1) with Zn(II), Ni(II), and Co(II) ions. The affinity of PEI cryogel to these ions is notably lower than to Cu(II) ions [13,21]; besides, in the case of a Co(II)-loaded monolith, it is important to treat cryogel with [Fe(CN)_6_]^4−^ ions as soon as possible to avoid the oxidation of Co(II) to Co(III), which binds to PEI irreversibly [13]. The level of the composite loading with inorganic sorbent using the Method I will be determined by the sorption capacity of the original PEI cryogel, which changes in the order Cu(II) > Zn(II) > Ni(II) > Co(II) (Figure S1, Supplementary Material), meaning that the composite cryogel with Zn(II) ferrocyanide would be the preferable option in terms of maximal loading with the sorption active component.

**Figure 2:**
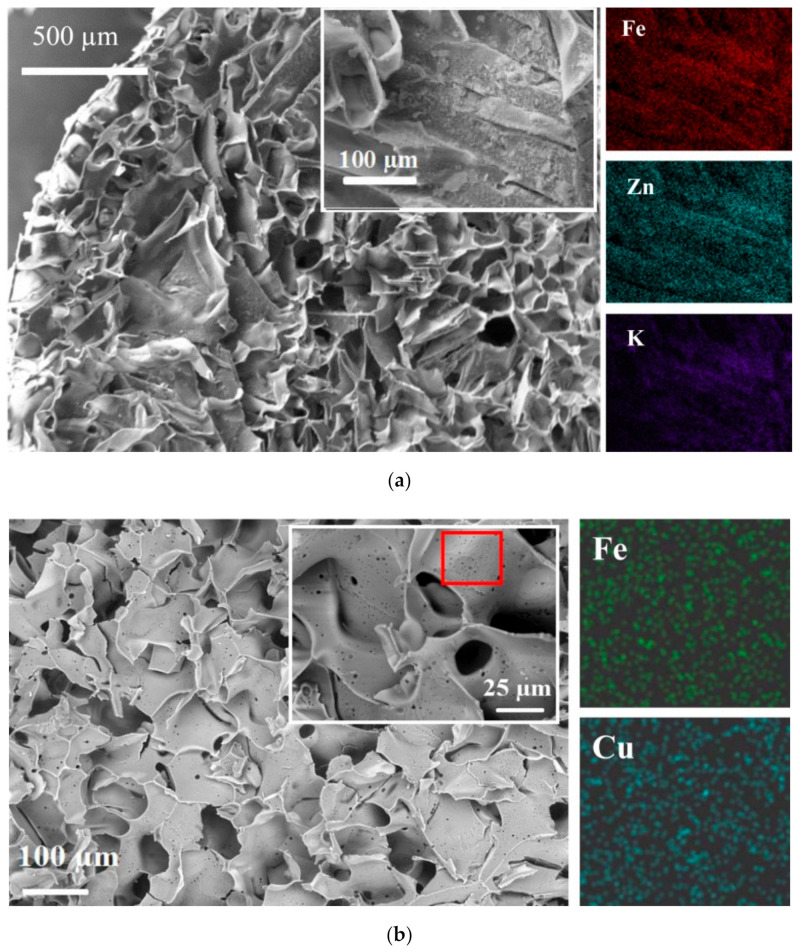
SEM images and EDX mapping for composite PEI cryogel containing Zn(II) ferrocyanide (**a**) and Cu(II) ferrocyanide (**b**) formed via Method I (Figure 1).

Figure 2 shows that in situ modification of PEI cryogel with ferrocyanides did not affect porous structure. The surface of the cryogel containing Zn(II) ferrocyanide (ZnFC) was covered with aggregates of nanocrystals, while the surface of the composite containing CuFC remained smooth. Smooth surfaces without a visible crystalline phase were also observed for composites containing Co(II) and Ni(II) ferrocyanides (CoFC and NiFC, respectively). However, EDX analysis in all cases confirmed the presence of homogeneously distributed Cu, Zn, Ni, or Co, and Fe at a M/Fe ratio close to the theoretical value of 2. Only for PEI/ZnFC cryogel was a significant amount of potassium detected by SEM-EDX, confirming the formation of the mixed potassium–zinc ferrocyanide (Table 1). Screening the sorption properties of the composites showed that only PEI/ZnFC cryogel was efficient for Cs^+^ ion uptake (Table 1).

Although the presence of alkali ions (mainly, K^+^) in ferrocyanides is often correlated with the enhancement of uptake kinetics and the increase of sorption capacities [8], good performance of ZnFC-based cryogel cannot be related directly to the formation of the mixed ferrocyanide in this case, since ion exchange of Cs^+^ ions with K^+^ is only one of the possible sorption mechanisms [12]. We showed earlier, for example, that Cu^2+^ ions were released upon efficient Cs^+^ sorption on composite polyallylamine/CuFC cryogel fabricated via the precipitation of the ex situ-formed CuFC colloids in the porous matrix [20]. SEM images of different areas of the monolith from top down and from the center to the periphery demonstrated homogeneous distribution of the ferrocyanide phase, which is an important advantage of the in situ fabrication method (Method I, Figure 1).

**Figure 3:**
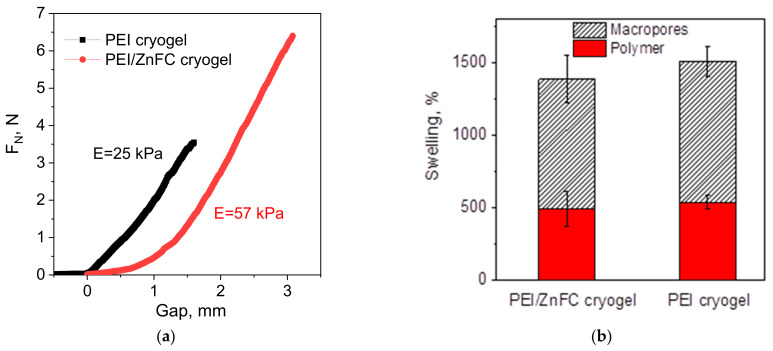
Uniaxial compression (**a**) and swelling (**b**) of the original PEI cryogel and composite PEI/ZnFC cryogel formed via Method I (Figure 1).

Comparison of the swelling behavior and stiffness of the original PEI cryogel and PEI/ZnFC composite (Figure 3) shows that the presence of the inorganic phase increased the stiffness of the cryogel, but had a minor effect on the overall swelling degree and contributions of free-flowing water (macropores) and bound water (polymer phase). As earlier reported for the composite cryogels [17,22], the introduction of inorganic particles into the porous polymer matrix could have a controversial effect on mechanical properties. Although the embedding of small TiO_2_ nanoparticles (6 nm) in the polymer walls notably increased compressive moduli [22], larger particles (25–27 nm) either improved [17] or worsened [22] mechanical properties of the composite.

**Figure 4:**
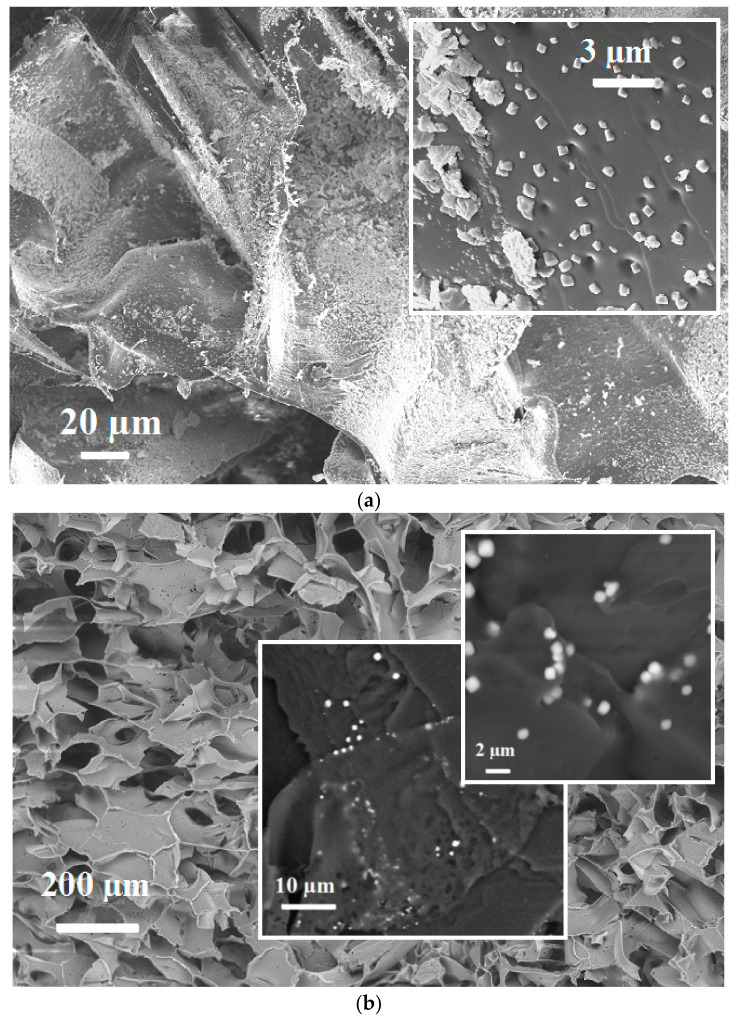
SEM images of the composite PEI/ZnFC cryogel fabricated via Method I (**a**) and Method II, upper part of the monolith (**b**).

The results obtained for the PEI/ZnFC composite here comply with the cryogel morphology. Since inorganic nanoparticles are located on the surface rather than embedded in the polymer, they do not have a drastic negative effect on cryogel stiffness, despite the large size of ZnFC nanoparticles (516 ± 146 nm) and the presence of aggregates of a size above 3 µm (Figure 4a). These aggregates also do not affect free water flow in the monolith due to the large pore size of the swollen PEI cryogel (128 ± 30 µm [23]). However, even a slightly decreased swelling degree of the composite cryogel can contribute to an increase in the Young modulus, since mechanical properties of cryogel significantly depend on water content. It should be noted that in our preliminary experiments aimed to control sorbent loading degree with PEI cross-linking density (i.e., the content of free amino groups capable of binding precursor metal ions), we found that highly cross-linked and less swellable cryogel was not applicable in columns, since the increase of stiffness after in situ formation of nanoparticles resulted either in a loss of permeability or in cryogel compression and formation of channels near the sorption column walls, meaning that the feeding solution went through these channels rather than through the cryogel bulk.

Method II (Figure 1), which assumes cross-linking of the polymer in dispersion containing ex situ-formed sorbent nanoparticles, is attractive due to its simplicity and possibility to control loading degree. At the same time, sedimentation of the sorbent nanoparticles during freezing and cross-linking stages can lead to the inhomogeneous distribution of the inorganic phase in a porous polymer matrix. To shorten the freezing time, the solutions were precooled, but, due to the low viscosity of PEI solution, sedimentation was difficult to avoid. For the sake of comparison between different fabrication methods, we have focused only on the composite containing Zn(II) ferrocyanide, which can be obtained by both methods. Figure 4b shows typical images for the composite fabricated via the Method II PEI porous structure with single ZnFC nanoparticles of average size 906 ± 165 µm, partially or completely embedded in the polymer matrix. At the same time, SEM-EDX analysis of different parts of the composite cryogel revealed inhomogeneous distribution of the inorganic phase from the center to the periphery and from top down, and the presence of the regions with large aggregates of ZnFC particles, which affected the integrity of the porous structure (Figure S2, Supplementary Materials).

Method III (Figure 1) is often used for the ligand-assisted fabrication of different nanoparticles, and, in comparison with Method II, this approach benefits from the stabilizing effect of the polymer at the stage of nanoparticle formation [24]. Due to the instability of transition-metal ferrocyanides in alkaline media, at the initial pH of PEI solution (pH~11), ZnFC nanoparticles that were formed immediately after addition of K_4_[Fe(CN)_6_] solution to the solution of Zn(II)-PEI complex were dissolved within a few dozen seconds. Adjusting the pH value to 7.4 allowed the formation of stable nanoparticle dispersion, than the cross-linking agent was added within 1 min and the dispersion was frozen. After thawing, the composite cryogel was permeable to the flow, but the SEM-EDX analysis showed drastic changes in morphology (Figure S2, Supplementary Materials). Although a typical macroporous structure of the PEI cryogel was preserved, large pores of a size from 1 to 15 µm appeared in the pore walls. The atomic ratio of Zn/Fe in the composite varied in a very broad range. In most spots, Zn was in non-stoichiometric excess, reaching a Zn/Fe atomic ratio of up to 15. The overall content of Zn and Fe was significantly lower than in composites obtained by the Methods I and II, although the same amounts of Zn(II) and [Fe(CN)_6_]^4−^ were introduced to all types of composites. It is likely that, under cryoconcentration conditions, PEI negatively affects the stability of initially formed ferrocyanide nanocrystals; therefore, being originally embedded to the polymer phase during the cross-linking step (7 days), they were dissolved and played the role of template rather than that of sorption active component.

### 2.2. Cs^+^ Sorption on PEI/ZnFC Composite Cryogels in Batch and Fixed Bed

**Figure 5:**
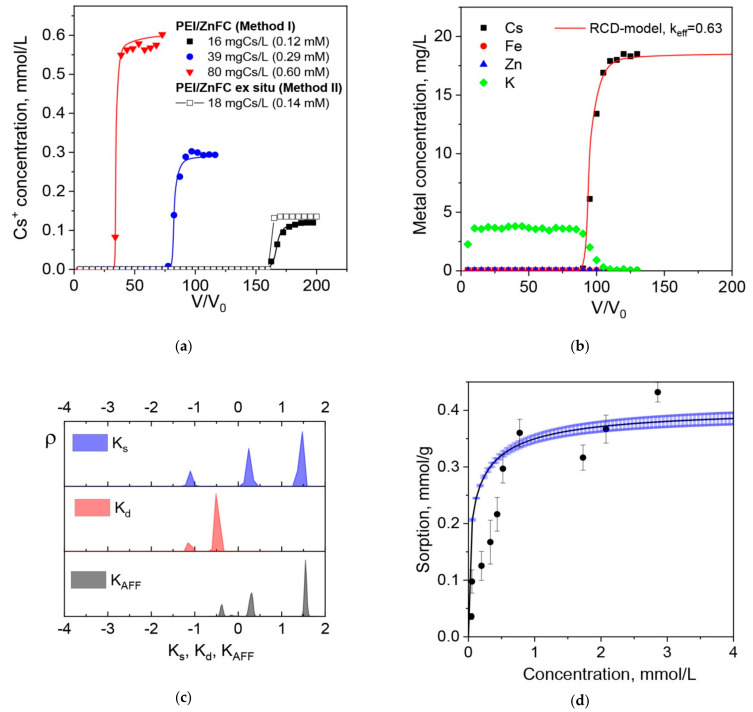
Breakthrough curves of Cs^+^ ions sorption on PEI/ZnFC composites fabricated via the Methods I and II, pH = 5, flow rate 100 BV/h, monolith column diameter was 0.48 cm, bed length was 6 cm: dots—experimental data; lines—RCD model (**a**). Breakthrough curve of Cs^+^ ions sorption on PEI/ZnFC composites (Method I) from the solution containing 19 mgCs/L at flow rate 145 BV/h and release profiles for zinc, potassium, and iron; column geometry is as in Figure 5a (**b**). 2D distributions of the Cs^+^ sorption sites of PEI/ZnFC cryogel (Method I) over the constants of sorption (K_s_) and desorption (K_d_) rates and affinity constants (K_AFF_) calculated using the rate-constant distribution (RCD) model (**c**). Isotherm of Cs^+^ ions sorption on PEI/ZnFC cryogel (Method I): dots—experimental data; line—calculations using RCD function (**d**).

Figure 5a shows the breakthrough curves for Cs^+^ ions sorption on PEI/ZnFC monoliths fabricated via Methods I and II. In all cases, residual Cs concentrations in the outlet solutions until breakthrough point were below the detection limit of AAS (<0.1 mg/L), so that Cs^+^ uptake efficiency was above 99.5%. The observed at high flow rate (100 BV/h = 5.53 m/h) steep breakthrough profiles are typical for sorption without significant mass transfer limitation, which makes PEI/ZnFC composite cryogel different from the earlier reported composite cryogels, which showed sloppy breakthrough curves without a horizontal region of high removal efficiency even at significantly lower superficial velocities [6,11].

The location of the ferrocyanide nanoparticles inside a polymer matrix (Method II) or at the interface (Method I) had no effect on the shape of the breakthrough curve under the sorption conditions shown in Figure 5a, although the sorption inactivity of nanoparticles embedded in a polymer matrix was reported for the composite cryogel for protein chromatography [18]. This proved that the diffusion of cesium ions in the polymer matrix was not the limiting stage of the sorption, complying with our previous study [23], which revealed a significant role of diffusion limitations only in the case of high-molecular-weight pollutant sorption on PEI cryogel. Thus, the sorption kinetics on ferrocyanide particles is expected to control the sorption performance of the PEI/ZnFC composite.

The release of K^+^ ions under dynamic conditions (Figure 5b) suggests that ion exchange between Cs^+^ ions in solution and K^+^ ions in the Zn_1.85_K_0.3_[Fe(CN)_6_] lattice significantly contributes to the sorption mechanism on PEI/ZnFC (Method I) composite. However, the maximal sorption capacity (~0.4 mmol/g) determined from the Cs^+^ sorption isotherm (Figure 5d) is higher than the dynamic sorption capacity (0.26 mmol/g) determined from breakthrough curves (Figure 5a) and the value 0.31 mmol/g, which can be expected from ZnFC chemical composition Zn_1.85_K_0.3_[Fe(CN)_6_] and Zn(II) content in monolith (Table 1). It is likely that the K^+^/Cs^+^ ion-exchange works for the fast sorption sites, which are active under dynamic conditions at high flow rates, but, under long equilibration times, other mechanisms or other sorption centers can contribute to the increased sorption capacity.

The presence of sorption sites with different sorption energies in the composite containing potassium–nickel ferrocyanide was hypothesized in [11], since the bi-site Langmuir equation provided a better fit to the experimental Cs^+^ sorption isotherm. Recently, we developed the extended rate-constant distribution (RCD) model for sorption in heterogeneous systems [13,14,21], which provides complete information about sorption properties for material with a continuous distribution of the sorption sites via the calculation of the RCD function from experimental data—sorption breakthrough (dynamics) or sorption kinetic (batch) curves experimentally obtained at different solid:liquid ratios or flow rates, and/or initial concentrations of the adsorbate. All experimental data are processed simultaneously to find the RCD function, which equally well describes a full set of experimental data. Using the RCD function, one can calculate several other distribution functions, including the 2D distribution of sorption sites over constants of sorption rate (ρ(K_s_) vs K_s_); 2D distribution of sorption sites over constants of desorption rate (ρ(K_d_) vs K_d_); and 2D affinity—distribution of sorption sites over affinity constants (ρ(K_AF_) vs K_AF_, K_AF_ = K_s_/K_d_ × Q_max_).

The application of this model to the analysis of Cs^+^ sorption breakthrough curves on the PEI/ZnFC composite (Method I, Figure 5a) enabled the calculation of several distribution functions, as shown in Figure 5c, and the identification of two types of Cs^+^ sorption centers with differences in sorption rate constants of more than 1 log unit. It is likely that the “slow” sites did not contribute significantly to the sorption under dynamic conditions (fixed bed), but could still be accounted for during calculation of the overall sorption capacity, since theoretical sorption capacity was in good agreement with experimental data (Figure 5d). It is worth mentioning that the investigation of sorption on composite cryogel (Method I) in batch under long equilibration times is complicated by the gradual destruction of the material with a release of nanoparticles into the solution, meaning that very thin discs of the composite were used instead of cryogel fines, which we used in metal ion sorption on the PEI cryogel to eliminate diffusion limitations [14]. The high number of experimental errors in the initial region of the sorption isotherm and the deviation between the RCD model and experimental data can be explained by this phenomenon.

Modeling the breakthrough curves for Cs^+^ ion sorption using the RCD function calculated for the PEI/ZnFC cryogel (Figure S3, Supplementary Materials) showed that the sorption rate constant (K_s_) is high enough to provide an efficient Cs^+^ uptake at flow rates of up to 500 BV/h. However, aside from the sorption rate, the important limiting factor is the mechanical stability of the composite, i.e., emergence of operational defects due to the disruption of the porous structure, as earlier observed for Zn(II) and Cu(II) sorption on PEI cryogel at flow rate 242 BV/h (superficial velocity of 13.3 m/h) [21]. Due to the higher stiffness of the composite (Figure 3b), the risks of such defects are increasing, e.g., Figure 5b shows that the breakthrough point at flow rate 145 BV/h was observed earlier than can be expected from the model, and the column efficiency was 63% of the theoretical value. The most pronounced effects of mechanical properties on sorption performance were observed for composites fabricated via Method II, most likely due to the inhomogeneous distribution of the inorganic phase. This composite showed significant differences in permeability depending on column geometry, was impermeable at flow rates above 100 BV/h and in sea water, while the composite fabricated via Method I was able to uptake Cs^+^ ions from sea water (Figure S4, Supplementary Materials), but with lower dynamic sorption capacity and release of ferrocyanide particles into the solution.

### 2.3. Cs-137 Uptake with PEI/ZnFC Composite

To avoid the risks of operational defects, the evaluation of the PEI/ZnFC cryogel (Method I) efficiency for Cs-137 radionuclide uptake was investigated at volumetric flow rates of up to 880 mL/h that in the column used (monolith cryogel volume of 8.48 cm^3^, diameter: height aspect ratio of 2.5; dry composite weight 0.7 g) corresponding to 105 BV/h and superficial velocity of 1.24 m/h.

**Figure 6:**
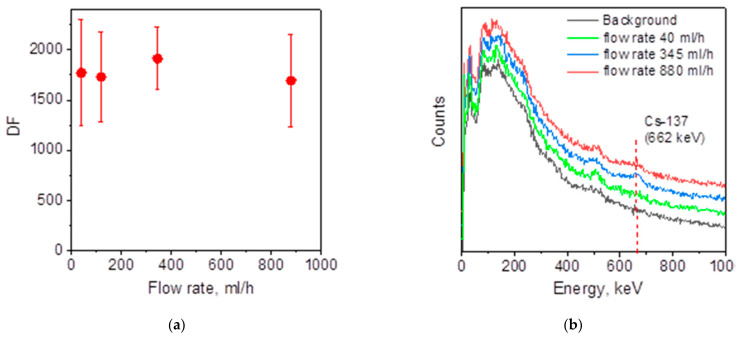
Dependence of decontamination factor (DF) on flow rate for Cs-137 removal with PEI/ZnFC cryogel fabricated via Method I (**a**) and γ-spectra of purified solutions and background (**b**).

Figure 6a shows that increasing flow rate from 40 to 880 mL/h had no systematic effect on the decontamination factor, which varied between 1600 and 1900. More precise DF calculations were limited by spectrometer sensitivity; however, in γ-spectra, very weak signals at 662 keV, corresponding to the emission of Cs-137, were detected only for the solutions decontaminated at flow rates of 345 and 880 mL/h, and no difference was observed between background and solution decontaminated at flow rate 40 mL/h (Figure 6b). This shows the significant advantages of PEI/ZnFC cryogel in comparison to porous chitin discs with immobilized K_2_Ni[Fe(CN)_6_] nanoparticles [11], which were efficient under static conditions, but failed to provide high DF in continuous mode without solution recirculation even at low superficial velocity (0.3 m/h).

We have tested the Cs-137 uptake using the same column geometry as in fixed-bed experiments (Figure 5a) at flow rates of 40, 80, and 120 mL/h corresponding to the superficial velocity 2.21, 4.42, and 6.63 m/h, respectively. In all cases, the residual γ-activity of the purified solutions corresponded to the background level. Thus, one can conclude that the efficient removal of Cs-137 with PEI/ZnFC fabricated with the Method I can be achieved at flow rates of at least up to 6.6 m/h.

This makes this composite cryogel attractive for POU application in flow-through devices in radioactively contaminated areas, primarily in household-type cartridges, e.g., jug water filters. High efficiency and high productivity in combination with small filter size, e.g., a relatively thin disc of the composite cryogel, as demonstrated above, are beneficial for in-field application to treat radioactively contaminated surface water, when other sources of drinking water are not available.

Cesium ion sorption on ferrocyanides is usually irreversible [12], although efficient cesium elution was reported in exceptional cases for mixed ferrocyanides [25]. Figure S5 (Supplementary Materials) that only about 25% of cesium was eluted with NaOH solution from the PEI/ZnFC cryogel fabricated via Method I at volumetric ratio eluent/column bed of 18. Virtually higher cesium elution efficiency can be reached with a larger volume of the eluent, but elution profile is not as steep as one expects for the reusable sorbents.

In POU application targeted to Cs-137 radionuclide removal, reusability of any kind of sorbents is not a crucial point, since regeneration would assume the production of the radioactive solution, which must be either processed or stored, while both options are not compatible with application in an emergency. It is more important that cryogel-based composites in dry form are light and, in comparison with matrices based on porous inorganic materials, can be efficiently compacted for safe storage until disposal. This feature can be also beneficial for analytical application, e.g., in Cs-137 ecological monitoring. High sorption efficiency at high flow rates allows cesium preconcentration from the surface water in field studies and collecting light and easy-to-transport samples, which can be further analyzed after oxidative destruction of the organic matrix or using non-invasive spectroscopic methods.

## 3. Materials and Methods

### 3.1. Materials

Branched polyethyleneimine (PEI) of an average molecular weight of 25 kDa was purchased from Alfa Aesar. 1,4-butanediol diglycidyl ether (DGEBD) was purchased from Sigma–Aldrich. All other reagents were of analytical grade.

### 3.2. Fabrication of the Composite Cryogels Containing Transition Metals Ferrocyanide

Composite cryogels were fabricated via three different methods (Figure 1). Method I (in situ): (i) monolith PEI cryogels were fabricated directly in the sorption columns via PEI (5% solution, pH 11) cross-linking with DGEBD at a molar ratio of 1:4 at −20 °C as described in [23]; (ii) M(NO_3_)_2_ solution (M=Cu(II), Zn(II), Ni(II), Co(II)) with M(II) concentration 100 mg/L was fed through the column (d = 4.8 mm, monolith volume = 1 mL) at a flow rate of 100 BV/h until complete saturation; to calculate the M(II) content in each monolith, the outlet M(II) concentration was controlled with atomic absorption spectroscopy (AAS) using a Solar M6 (Thermo Scientific, Waltham, MA, USA) device; (iii) after washing the monolith with distilled water, 0.01 M K_4_[Fe(CN)_6_] solution was fed through the column at a flow rate of 8 BV/h, the required volume of K_4_[Fe(CN)_6_] solution was calculated to assure molar excess in respect to the M(II) content in the monolith. The obtained composites were thoroughly washed with distilled water and kept swollen until used. For the sorption experiments in batch and morphology investigations, cryogels were removed from the column and air-dried.

Method II (ex situ): (i) 0.2 M solutions of Zn(NO_3_)_2_ and K_4_[Fe(CN)_6_] were mixed at molar ratio of 2:1 and left under constant stirring for 18 h; (ii) water and 10% PEI solution was added to the dispersion of Zn(II) ferrocyanide nanoparticles under intensive stirring for 1 min, so that final concentration of PEI is 5% and the Zn(II) content is 110 mg/g of PEI, then DGEBD was added at a molar ratio to PEI of 1:4; (iii) after cross-linker addition solutions were immediately placed into syringes and kept frozen for 7 days at −20 °C.

Method III: (i) 0.2 M solution of Zn(NO_3_)_2_ was added to the 10% solution of PEI (pH 7.40) to provide the Zn(II) content of 110 mg/g of PEI; after stirring during 30 min 0.2 M solution of K_4_[Fe(CN)_6_] was added at the molar ratio to Zn(II) of 1:2 with subsequent DGEBD addition at a molar ratio to PEI of 1:4. Solutions were immediately placed into syringes and kept frozen for 7 days at −20 °C.

### 3.3. Characterization of Morphology and Composition of the Cryogels

Morphology investigations and elemental analysis of the composite cryogels were performed using a Hitachi TM3000 scanning electron microscope (SEM) equipped with a Bruker Quantax 70 energy dispersive X-ray (EDX) spectrometer at an accelerating voltage of 15 kV (Figure 2b, Figure 4b, and Figure S2). Alternatively, the surface of the sample was sprayed with Cr using Q150T ES (Quorum Technologies, Lewes, UK), the coating thickness was 10 nm, and the morphology of cryogels was studied using a Zeiss SIGMA 300 VP microscope (Carl Zeiss Group, Oberkochen, Germany) with SE2 secondary electron detector. SEM images were obtained at an accelerating voltage of 20 kV (Figure 4a), and the elemental composition was studied using an X-ray (EDX) spectrometer X-MAX (Bruker, Billerica, USA) mounted on a scanning electron microscope Evo 40 (Oxford Instruments, Abingdon, UK) at an accelerating voltage of 20 kV (Figure 2a). The elemental composition (Table 1) was calculated for the sum of elements (Fe, K, and M, where M=Cu, Zn, Ni, Co).

### 3.4. Characterization of Cryogels Stiffness and Swelling Behavior

The Young’s modulus (E) was calculated from the linearized dependence of the normal force (FN) vs gap recorded during uniaxial compression of the cylindrically shaped cryogels at a constant speed of 0.01 mm/s using a Physica MCR 301 rheometer (Anton Paar GmbH, Graz, Austria), as follows:

(1) E=l0·FNS·Δl

where F_N_ is the normal force, l_0_ is the initial sample height, Δl is the change in the sample height, and S is the area of the material.

The cylinders of PEI cryogel and PEI/Zn(II) ferrocyanide cryogel were 25 mm in diameter and 6–8 mm in height.

Swelling of cryogels was determined from the difference in weights of the completely swollen and dried (18 h, 90 °C) material. The contribution of free-flowing water (macropores) to the swelling was determined from the difference in weights of the completely swollen and finger-squeezed cryogel.

### 3.5. Investigations of the Sorption Properties of the Composite Cryogels in Batch

The efficiency of the Cs^+^ ion uptake by the composite cryogels obtained according to Method I with Cu(II), Zn(II), Ni(II), and Co(II) ferrocyanides (PEI/CuFC, PEI/ZnFC, PEI/NiFC, PEI/CoFC, respectively) was determined in batch as follows: 5 mg of the composite cryogel was shaken for 24 h with 5 mL of CsCl solution containing 20 mgCs/L (pH~6).

The isotherm of Cs^+^ sorption on the composite cryogel PEI/ZnFC fabricated via Method I was investigated at T = 25 °C, the sorbent:solution ratio 1:1000, the contact time of 72 h with constant agitation at 200 rpm using a Biosan PSU-20i orbital shaker (Riga, Latvia). For this experiment, the monolith PEI/ZnFC cryogel of a diameter of 4.8 mm was cut to 1–2 mm discs to reduce diffusion limitations. The adsorbed amounts were calculated using the difference in Cs initial and equilibrium concentrations determined by AAS using a Solar M6 (Thermo Scientific, Waltham, MA, USA) device.

### 3.6. Investigations of the Sorption Properties of PEI/ZnFC Composite Cryogels in Fixed Bed

Dynamics of Cs^+^ ion sorption on PEI/ZnFC cryogels fabricated via Methods I and II was studied by feeding CsCl solutions containing 20, 40, or 80 mgCs/L (pH~6) through 1 mL of the monolith (d = 0.48 cm, h = 6–6.2 cm) at a flow rate of 100 or 145 BV/h using a peristaltic pump. The filtrate was collected every 5 mL and analyzed for cesium using AAS. In selected experiments, zinc, iron, and potassium concentrations in the outlet solutions were also determined by AAS. The sea water for investigation of the Cs^+^ sorption dynamics was collected in Amur bay, Sea of Japan (43.19842° N, 131.919491° E), filtered through a “blue ribbon” paper filter, and spiked with CsCl solution to yield Cs concentration of 48 mg/L.

Removal of Cs-137 radionuclide using monolith PEI/ZnFC cryogel fabricated via Method I (d = 3 cm, h = 1.2 cm) was investigated from 0.001 M NaNO_3_ solution spiked with Cs-137 (initial activity was 265 kBq/L). Solution containing Cs-137 was fed through the monolith cryogel using a peristaltic pump with the flow rate increased stepwise from 40 mL/h to 880 mL/h. At each step 20 mL was collected to record gamma (γ) spectra with a step of 3 keV and to determine the specific γ-activity using an AT 1315 Gamma–Beta spectrometer with a 63 × 63 mm NaI (Tl) detector (ATOMTEX, Minsk, Belarus). The measurement time was set to the maximum (10 h); activity of 0.001 M NaNO_3_ solution without Cs-137 tracer was taken as a background. Decontamination factor (DF) corresponding to each flow rate was calculated as follows:

(2) $$DF=\frac{A_{0}}{A}$$

where *A* and *A_0_* are the ^137^Cs γ-activities (specific activity, Bq/L) of outlet and inlet solutions, respectively.

## 4. Conclusions

Three different approaches to the fabrication of polymer–inorganic composite sorbent have been investigated to find an optimum method to produce highly porous monolith sorbent for application in radioactively contaminated areas for Cs^+^ ion removal using a point-of-use technology.

The fabrication methods were based on the in situ formation of ferrocyanide nanoparticles via sequential ion-exchange steps in pre-formed polyethyleneimine cryogel (“in situ composite”, Method I), immobilization of ex situ synthesized ferrocyanide nanoparticles to cryogel at the cross-linking stage (“ex situ composite”, Method II), and via sequential ion-exchange steps in polyethyleneimine solution followed by polymer cross-linking at subzero temperature(“in situ in solution composite”, Method III).

We have shown that “in situ composite” containing mixed zinc–potassium ferrocyanide had significant advantages over “ex situ composite” in terms of homogeneity of inorganic phase distribution, reproducibility, and permeability to the flow. “In situ in solution composite” did not show sorption activity due to the dissolution of the initially formed inorganic phase.

In comparison with the earlier reported porous materials for cesium ion uptake, the fabricated supermacroporous composite containing mixed ferrocyanide Zn_1.85_K_0.33_[Fe(CN)_6_] demonstrated a very steep breakthrough profile at a superficial velocity of up to ~8 m/h with a dynamic adsorption capacity of 0.29 mmolCs/g and efficiency of Cs^+^ uptake >99.5%. For the treatment of Cs-137 spiked solution with initial specific activity of 265 kBq/L, a decontamination factor of 1600–1900 was reached at a superficial velocity of up to 6.6 m/h, thus allowing a reduction of the γ-activity to the background level. In comparison with composites based on porous inorganic matrices, composite cryogels are light and compact in the dry state and much easier to dispose of. They can also be applied for the analytical preconcentration of Cs-137 radionuclides from surface water.

## Figures and Tables

**Table 1:** Elemental analysis (SEM-EDX data) and sorption efficiency of the composite cryogel containing transition-metal ferrocyanides (FC).

Composite Cryogel	M ^1^, mg/g	M, at %	Fe, at %	K, at %	Ferrocyanide Composition	Efficiency of Cs^+^ Uptake ^2^, %
PEI/CuFC	134	66.70	33.28	0.02	Cu_2_[Fe(CN)_6_]	2
PEI/ZnFC	114	58.21	31.35	10.44	Zn_1.85_K_0.3_[Fe(CN)_6_]	84
PEI/NiFC	59	65.49	32.82	1.69	Ni_1.99_K_0.02_[Fe(CN)_6_]	5
PEI/CoFC	29	65.31	32.77	1.92	Co_1.99_K_0.02_[Fe(CN)_6_]	0

^1^ Content of Cu(II), Zn(II), Ni(II), or Co(II) in composite cryogel, ^2^ Efficiency of Cs^+^ uptake from solution containing 20 mgCs/L (pH~6) at solid: liquid ratio 1:1000.

## Data Availability

Row sorption data are available from the authors upon request.

## Supplementary Materials

The following are available online. Figure S1: Isotherms of metal ions sorption on PEI cryogel cross-linked with DGEBD at molar ratio DGEEB:PEI 1:4, pH = 5, T = 25 °C, Solid:Liquid ratio 1:1000, contact time 72 h. Figure S2: SEM image of the composite PEI/ZnFC cryogels fabricated via Method III (a) and Method II (b,c). Figure S3: Theoretical (RCD model) breakthrough curves of Cs^+^ ions sorption on the PEI/ZnFC composite fabricated via Method I, 19 mg Cs/L (0.14 mM), monolith column diameter was 3 cm, bed length was 1.2 cm. Figure S4: Breakthrough curves of the Cs^+^ ions sorption on PEI/ZnFC composite fabricated via Method I and release profiles for zinc and iron, sea water, 48 mg Cs/L, flow rate 100 BV/h, monolith column diameter was 0.48 cm, bed length was 6 cm: dots—experimental data; dash line—RCD model. Figure S5: Elution of Cs^+^ ions from PEI/ZnFC composite fabricated via Method I using 0.1 M NaOH solution, flow rate 8 BV/h.

## Sample Availability

Samples of the materials compounds are available from the authors upon request providing responsibility of the Requester on custom regulations and shipment costs.

## References

[1] Dankovich T.A., Smith J.A. (2014). Incorporation of copper nanoparticles into paper for point-of-use water purification. Water Res..

[2] Ehdaie B., Krause C., Smith J.A. (2014). Porous ceramic tablet embedded with silver nanopatches for low-cost point-of-use water purification. Environ. Sci. Technol..

[3] Fan M., Gong L., Huang Y., Wang D., Gong Z. (2018). Facile preparation of silver nanoparticle decorated chitosan cryogels for point-of-use water disinfection. Sci. Total Environ..

[4] Ma C.B., Du Y., Du B., Wang H., Wang E. (2018). Investigation of an eco-friendly aerogel as a substrate for the immobilization of MoS 2 nanoflowers for removal of mercury species from aqueous solutions. J. Colloid Interface Sci..

[5] Lu X., Huangfu X., Ma J. (2014). Removal of trace mercury(II) from aqueous solution by in situ formed Mn-Fe (hydr)oxides. J. Hazard. Mater..

[6] Suresh Kumar P., Önnby L., Kirsebom H. (2013). Arsenite adsorption on cryogels embedded with iron-aluminium double hydrous oxides: Possible polishing step for smelting wastewater?. J. Hazard. Mater..

[7] Vincent C., Barré Y., Vincent T., Taulemesse J.M., Robitzer M., Guibal E. (2015). Chitin-Prussian blue sponges for Cs(I) recovery: From synthesis to application in the treatment of accidental dumping of metal-bearing solutions. J. Hazard. Mater..

[8] Vincent T., Vincent C., Guibal E. (2015). Immobilization of Metal Hexacyanoferrate Ion-Exchangers for the Synthesis of Metal Ion Sorbents—A Mini-Review. Molecules.

[9] Vincent T., Vincent C., Barré Y., Guari Y., Le Saout G., Guibal E. (2014). Immobilization of metal hexacyanoferrates in chitin beads for cesium sorption: Synthesis and characterization. J. Mater. Chem. A.

[10] Zong Y., Zhang Y., Lin X., Ye D., Qiao D., Zeng S. (2017). Facile synthesis of potassium copper ferrocyanide composite particles for selective cesium removal from wastewater in the batch and continuous processes. RSC Adv..

[11] Vincent C., Hertz A., Vincent T., Barré Y., Guibal E. (2014). Immobilization of inorganic ion-exchanger into biopolymer foams—Application to cesium sorption. Chem. Eng. J..

[12] Haas P.A. (1993). A Review of Information on Ferrocyanide Solids for Removal of Cesium from Solutions. Sep. Sci. Technol..

[13] Golikov A., Malakhova I., Azarova Y., Eliseikina M., Privar Y., Bratskaya S. (2020). Extended Rate Constant Distribution Model for Sorption in Heterogeneous Systems. 1: Application to Kinetics of Metal Ion Sorption on Polyethyleneimine Cryogels. Ind. Eng. Chem. Res..

[14] Malakhova I., Golikov A., Azarova Y., Bratskaya S. (2020). Extended Rate Constants Distribution (RCD) Model for Sorption in Heterogeneous Systems: 2. Importance of Diffusion Limitations for Sorption Kinetics on Cryogels in Batch. Gels.

[15] Lozinsky V. (2018). Cryostructuring of Polymeric Systems. 50 Cryogels and Cryotropic Gel-Formation: Terms and Definitions. Gels.

[16] Otero-González L., Mikhalovsky S.V., Václavíková M., Trenikhin M.V., Cundy A.B., Savina I.N. (2020). Novel nanostructured iron oxide cryogels for arsenic (As(III)) removal. J. Hazard. Mater..

[17] Shu Y., Huang R., Wei X., Liu L., Jia Z. (2017). Pb(II) Removal Using TiO_2_-Embedded Monolith Composite Cryogel as an Alternative Wastewater Treatment Method. Water Air Soil Pollut..

[18] Yao K., Yun J., Shen S., Wang L., He X., Yu X. (2006). Characterization of a novel continuous supermacroporous monolithic cryogel embedded with nanoparticles for protein chromatography. J. Chromatogr. A.

[19] Bratskaya S., Privar Y., Slobodyuk A., Shashura D., Marinin D., Mironenko A., Zheleznov V., Pestov A. (2019). Cryogels of Carboxyalkylchitosans as a Universal Platform for the Fabrication of Composite Materials. Carbohydr. Polym..

[20] Malakhova I., Privar Y., Parotkina Y., Mironenko A., Eliseikina M., Balatskiy D., Golikov A., Bratskaya S. (2020). Rational design of polyamine-based cryogels for metal ion sorption. Molecules.

[21] Golikov A., Malakhova I., Privar Y., Parotkina Y., Bratskaya S. (2020). Extended Rate Constant Distribution Model for Sorption in Heterogeneous Systems: 3. From Batch to Fixed-Bed Application and Predictive Modeling. Ind. Eng. Chem. Res..

[22] Suwanchawalit C., Patil A.J., Kumar R.K., Wongnawa S., Mann S. (2009). Fabrication of ice-templated macroporous TiO_2_-chitosan scaffolds for photocatalytic applications. J. Mater. Chem..

[23] Malakhova I., Privar Y., Azarova Y., Eliseikina M., Golikov A., Skatova A., Bratskaya S. (2020). Supermacroporous monoliths based on polyethyleneimine: Fabrication and sorption properties under static and dynamic conditions. J. Environ. Chem. Eng..

[24] Rozenberg B.A., Tenne R. (2008). Polymer-assisted fabrication of nanoparticles and nanocomposites. Prog. Polym. Sci..

[25] Tokar’ E., Zemskova L., Tutov M., Tananaev I., Dovhyi I., Egorin A. (2020). Development and practical evaluation of the scheme for 137Cs concentrating from seawater using chitosan and mixed ferrocyanides of Zn-K and Ni-K. J. Radioanal. Nucl. Chem..

