# Supplementary material for: Composite Zn(II) Ferrocyanide/Polyethylenimine Cryogels for Point-of-Use Selective Removal of Cs-137 Radionuclides
Source: Molecules. 2021 Jul 29;26(15):4604. doi: 10.3390/molecules26154604 (PMC8347221; doi:10.3390/molecules26154604)
Supplement: Supplementary file 1 [file molecules-26-04604-s001.zip › molecules-1301010-supplementary.pdf]

SUPPLEMENTARY MATERIALS

Composite Zn(II) Ferrocyanide/Polyethylenimine Cryogels for Point-of-Use Selective Removal of Cs-137Radionuclides

Irina Malakhova [1], Yuliya Parotkina [1], Marina Palamarchuk [1], Marina Eliseikina [2], AleksandrMironenko [1], Alexey Golikov [1], and Svetlana Bratskaya [1,*]

[1] Institute of Chemistry, Far Eastern Branch of Russian Academy of Sciences, 159, prosp.100-letiya Vladivostoka,690022 Vladivostok, Russia

[2] A.V. Zhirmunsky National Scientific Center of Marine Biology, Far Eastern Branch of Russian Academy of Sciences, 17, Palchevskogo street, 690041 Vladivostok, Russia

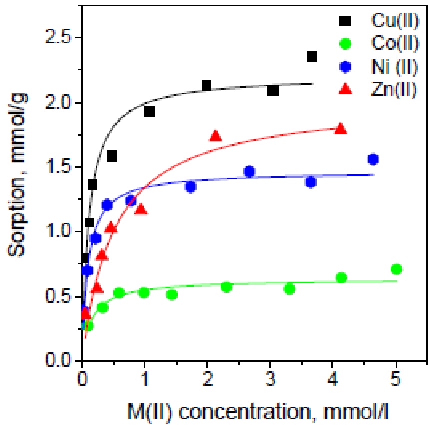

Figure S1. Isotherms of metal ions sorption on PEI cryogel cross-linked with DGEBD at molarratio DGEEB:PEI 1:4, pH = 5, T = 25°C, Solid:Liquid ratio 1:1000, contact time 72 h.

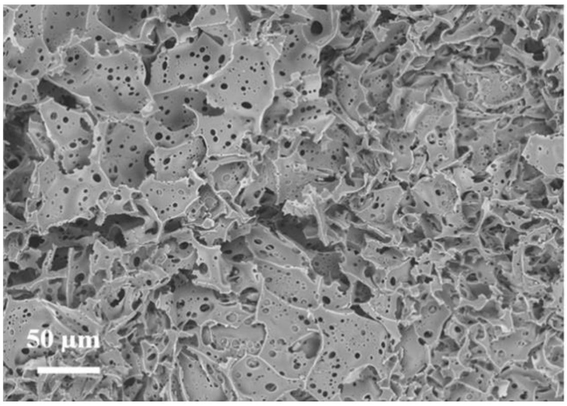

(a)

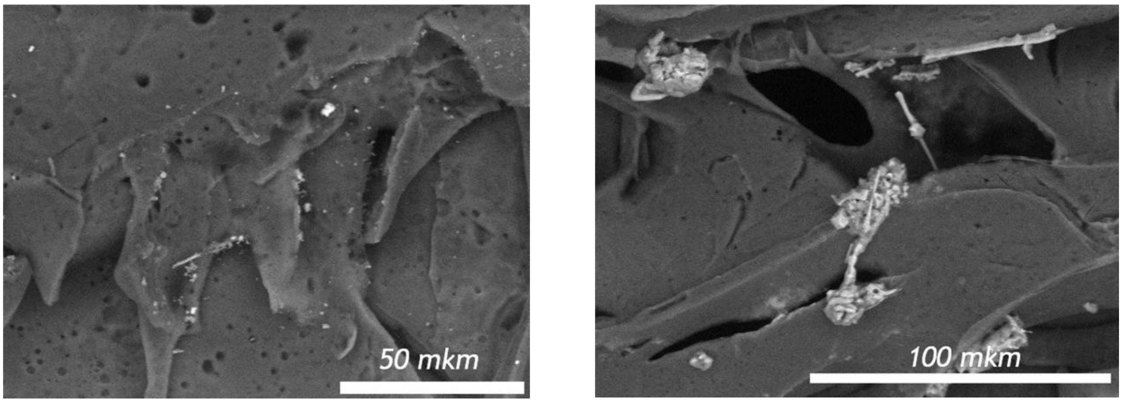

(b) **(c)**

Figure S2. SEM image of the composite PEI/ZnFC cryogels fabricated via Method III (**a**) andMethod II (**b**, **c**).

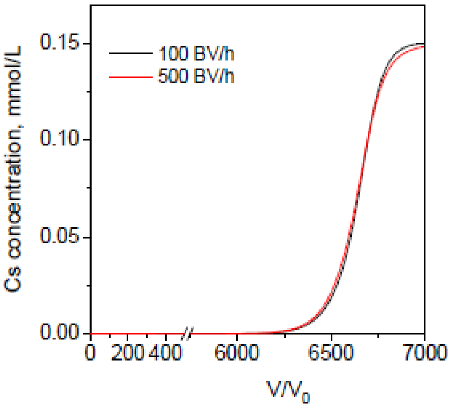

Figure S3. Theoretical (RCD-model) breakthrough curves of Cs^+ ions sorption on PEI/ZnFC composite fabricated via Method I, 19 mgCs/L (0.14mM), monolith column diameter was 3 cm,bed length was 1.2 cm.

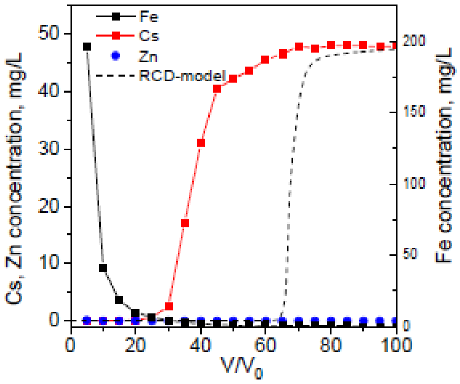

Figure S4. Breakthrough curves of Cs^+ ions sorption on PEIZnFC composite fabricated via Method I and release profiles for zinc and iron, sea water, 48 mgCs/L, flow rate 100 BV/h, monolith column diameter was 0.48 cm, bed length was 6 cm: dots - experimental data, dash line - RCD-model **(a)**. Breakthrough curve of Cs^+ ions sorption on PEI/ZnFC composites (Method I) from the solution

containing 19 mgCs/L at flow rate 145 BV/h and release profiles forzinc, potassium and iron; column geometry as in Fig.5a.

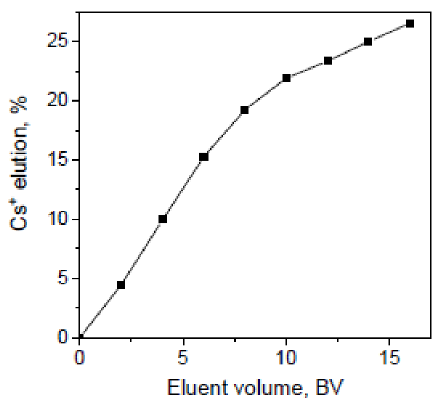

Figure S5. Elution of Cs^{+} ions from PEI/ZnFC composite fabricated via Method I using 0.1M NaOH solution, flow rate 8 BV/h.
